# miRNA-target prediction based on transcriptional regulation

**DOI:** 10.1186/1471-2164-14-S2-S3

**Published:** 2013-02-15

**Authors:** Toyofumi Fujiwara, Tetsushi Yada

**Affiliations:** 1 INTEC Inc., 1-3-3 Shinsuna, Koto-ku, Tokyo 136-8637, Japan; 2 Graduate School of Informatics, Kyoto University, Yoshida Honmachi, Sakyo-ku, Kyoto 606-8501, Japan

## Abstract

**Background:**

microRNAs (miRNAs) are tiny endogenous RNAs that have been discovered in animals and plants, and direct the post-transcriptional regulation of target mRNAs for degradation or translational repression via binding to the 3'UTRs and the coding exons. To gain insight into the biological role of miRNAs, it is essential to identify the full repertoire of mRNA targets (target genes). A number of computer programs have been developed for miRNA-target prediction. These programs essentially focus on potential binding sites in 3'UTRs, which are recognized by miRNAs according to specific base-pairing rules.

**Results:**

Here, we introduce a novel method for miRNA-target prediction that is entirely independent of existing approaches. The method is based on the hypothesis that transcription of a miRNA and its target genes tend to be co-regulated by common transcription factors. This hypothesis predicts the frequent occurrence of common *cis*-elements between promoters of a miRNA and its target genes. That is, our proposed method first identifies putative *cis*-elements in a promoter of a given miRNA, and then identifies genes that contain common putative *cis*-elements in their promoters. In this paper, we show that a significant number of common *cis*-elements occur in ~28% of experimentally supported human miRNA-target data. Moreover, we show that the prediction of human miRNA-targets based on our method is statistically significant. Further, we discuss the random incidence of common *cis*-elements, their consensus sequences, and the advantages and disadvantages of our method.

**Conclusions:**

This is the first report indicating prevalence of transcriptional regulation of a miRNA and its target genes by common transcription factors and the predictive ability of miRNA-targets based on this property.

## Background

microRNAs (miRNAs) are tiny endogenous RNAs which occur in animals and plants and that direct the post-transcriptional regulation of target mRNAs for degradation or translational repression via binding to the 3'UTRs and the coding exons [1-4]. More than 1,500 miRNA genes have been identified in the human genome [5]. Computational predictions have shown that miRNAs may directly regulate 20-30% of protein-coding genes [6,7], and, on average, each miRNA can regulate the expression of several hundred genes [8]. Therefore, miRNAs are regarded as important regulators for cell differentiation, proliferation/growth, mobility, and apoptosis [9-11].

To gain insight into the biological role of miRNAs, it is essential to identify the full repertoire of mRNA targets (target genes). A number of computer programs have been developed for miRNA-target prediction [12]. These programs essentially perform two steps. First, they identify potential binding sites in 3'UTRs, which are recognized by the seed region of a given miRNA according to specific base-pairing rules. The seed region is defined as the consecutive stretch of 7 nucleotides starting from either the first or the second nucleotide at the 5' end of a miRNA. Note that they do not take potential binding sites in coding exons into consideration. Second, they evaluate cross-species conservation of the potential binding sites, and regard mRNAs with high conservation as putative target genes. This step successfully reduces many false positive predictions. However, it is increasingly evident that many non-conserved binding sites are also functional [13].

Accordingly, several programs that do not rely on cross-species conservation have been developed. These programs employ novel features in addition to base-pairing rules in seed regions. Kim *et al*. [14] and Yousef *et al*. [15] introduced various types of features observed in downstream seed regions (out-seed regions), e.g. structural, thermodynamic and positional features. Robins *et al*. [16] and Kertesz *et al*. [17] incorporated mRNA secondary structure as a measure of accessibility to miRNA-target binding sites in their prediction programs. Wang & Naqa [18] and Gennarino *et al*. [19] proposed integration of gene expression data into their prediction programs. Nonetheless, almost all the programs had region-limited view of miRNA activity, that is, they focused on potential binding sites in 3'UTRs of mRNAs only.

**Figure 1:**
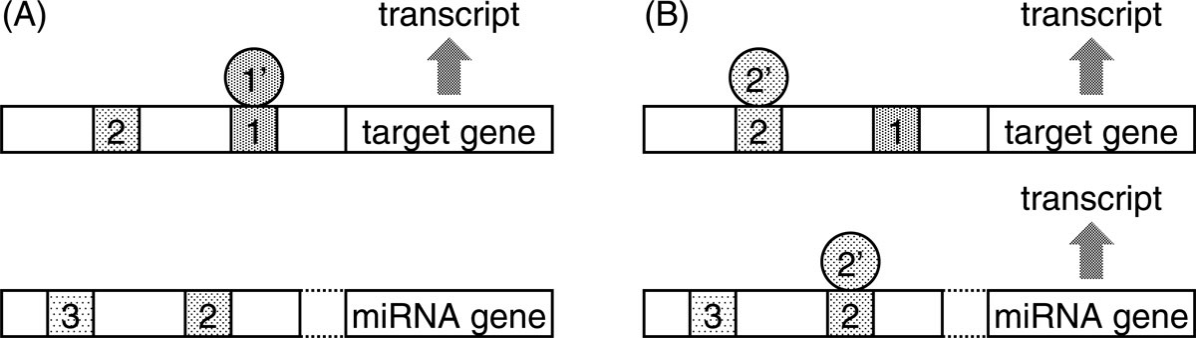
**Schematic diagram of our hypothesis**. Filled rectangles indicate *cis*-elements in promoters. Circles indicate transcription factors, and transcription factor *x' *binds to *cis*-element *x*. *Cis*-element 2 is common in both miRNA and target gene promoters, while *cis*-elements 1 and 3 are specific in target and miRNA genes, respectively. In this figure, all transcription factors are regarded as activators. (A) In a case that transcription factor 1' binds to *cis*-element 1, only the target gene is transcribed. (B) In a case that transcription factor 2' binds to *cis*-element 2, both the miRNA and the target genes are transcribed. The miRNA subsequently downregulates the expression of the target gene after several transportation and processing steps. In a case that transcription factor 3' binds to *cis*-element 3, the expression of genes whose promoters contain *cis*-element 3 will be downregulated by the miRNA.

We introduce here a novel method for miRNA-target prediction that is completely different from existing approaches. The method is based on the hypothesis that transcription of a miRNA and its target genes tends to be co-regulated by common transcription factors (Figure 1). This hypothesis is supported by several lines of evidence, such as the observation that the miRNA *miR-17-5p *and its target gene *E2F1 *are both transcriptionally activated by *c-Myc *in human cells [20]. Marco *et al*. [21] reported that pairs of genes with shared *cis*-element showed, on average, a higher degree of co-expression than those with no common *cis*-element (however, the reverse did not hold true). Therefore, this hypothesis predicts that common *cis*-elements may occur occasionally between promoters of a miRNA and its target genes. That is, the proposed method first identifies putative *cis*-elements in the promoter of a given miRNA, and then identifies genes that have similar putative *cis*-elements in their promoters. We adopted human as a model organism because human has the most comprehensive miRNA-target data and genome annotations [22-24].

In terms of genomic organization, miRNAs can be categorized into two classes, namely, intragenic and intergenic miRNAs [25]. Intragenic miRNAs are located within other transcriptional units (host genes). Rodriguez *et al*. [26] proposed that such miRNAs are transcribed in parallel with their host genes, suggesting that they share promoters with their host genes. In contrast, intergenic miRNAs are located between other transcriptional units and therefore have their own transcriptional units and promoters. Lee *et al*. [27] verified that they are first transcribed as long primary transcripts (pri-miRNAs) by RNA polymerase II. These long pri-miRNAs are then processed into pre-miRNAs and mature miRNAs. Intergenic miRNAs occasionally form a cluster, and these can be simultaneously transcribed as a single polycistronic transcript [28]. Short distances between consecutive intergenic miRNA loci are hallmarks of polycistronic transcription.

We discuss here two questions. (1) Are there common *cis*-elements between promoters of a miRNA and its target genes? (2) Is it possible to predict miRNA-target genes based on common *cis*-elements? First, we found that a significant number of common *cis*-elements were observed in ~28% of experimentally supported miRNA-target data. Second, we demonstrate the statistical significance of the predictive ability of our method. Finally, we discuss the random background resulting from common *cis*-elements, consensus sequences of these elements, and the advantages and disadvantages of our method. This is the first report indicating prevalent transcriptional regulation of a miRNA and its target genes by common transcription factors and the potential to predict miRNA targets based on this property.

## Results and discussion

### Finding common cis-elements

For each set of miRNA-target data, we detected a set of common *cis*-elements between promoters of the miRNA and its target gene, and evaluated its statistical significance. As a result, we observed at least one common *cis*-element in 73 (73/97) of the intragenic miRNA-target data and 62 (62/110) of the intergenic miRNA-target data. Among these, 32 (32/97) of the intragenic miRNA-target data and 25 (25/110) of the intergenic miRNA-target data were found to be statistically significant. That is, we observed a statistically significant number of common *cis*-elements in 57 (57/207) of the miRNA-target data. This corresponds to 27.5% of the data, and clearly shows the prevalence of transcriptional regulation of a miRNA and its target gene by common transcription factors. Although pairs of genes with common *cis*-elements show, on average, a higher degree of co-expression than those without, gene pairs with higher degrees of expression correlation do not have significantly greater numbers of common *cis*-elements [21]. Thus, there is a possibility that a greater fraction of the miRNA-target data is actually co-expressed.

#### Why were common cis-elements so frequently observed?

**Figure 2:**
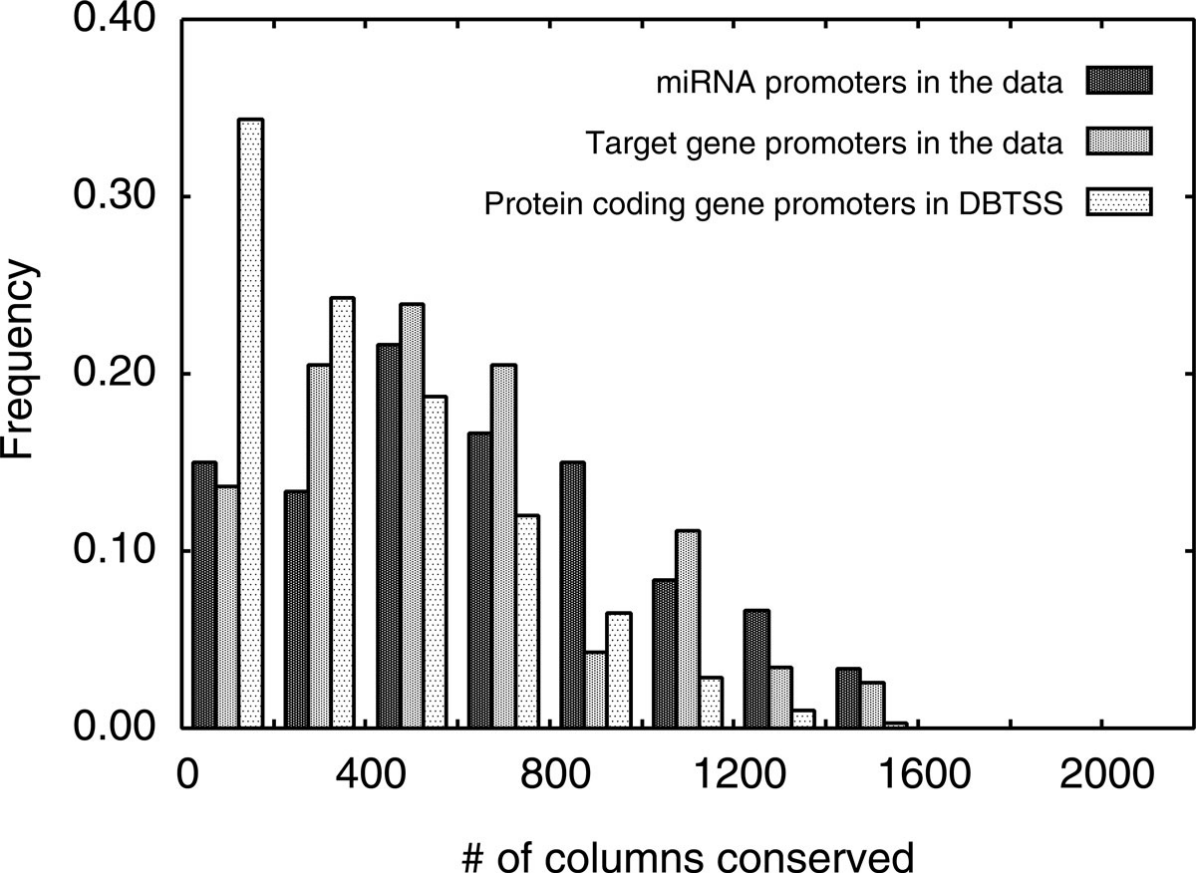
**Distribution of number of conserved columns between the five mammals (human, chimp, mouse, rat and dog) in multiple sequence alignments of respective promoters**. The multiple sequence alignments, which were 2,200 bp long, were obtained from the UCSC Genome Browser.

We found that promoters of miRNAs and target genes were well conserved (Figure 2). On average, 641 and 581 columns were conserved in multiple sequence alignments of miRNA and target gene promoters, respectively. In contrast, only an average of 357 columns were conserved in multiple sequence alignments of promoters of human protein coding genes from DBTSS. This reflects an enrichment of functional sites in promoters of miRNAs and target genes, and may suggest more complex regulation of these promoters at the transcription level.

#### Are there consensus sequences between common cis-elements?

**Figure 3:**
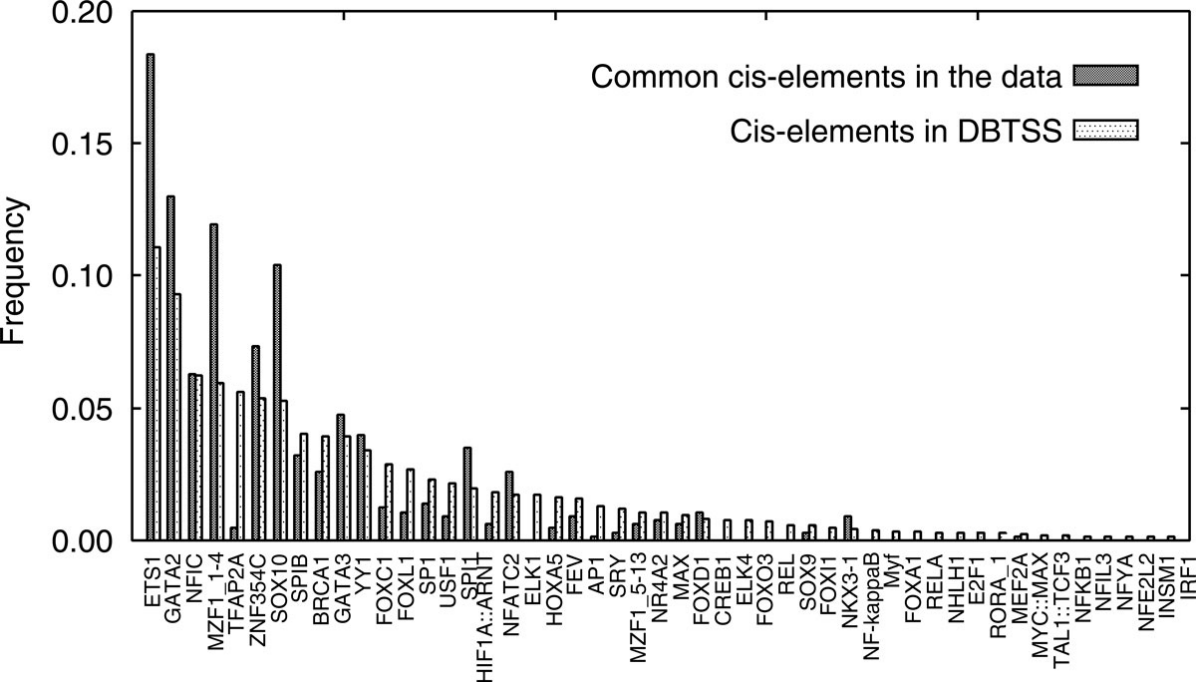
**Two frequency distributions of JASPAR matrix models to which we assigned the common *cis*-elements and *cis*-elements from DBTSS protein-coding gene promoters**. JASPAR matrix models are ranked by the latter frequencies, and are sorted in ascending rank order along the *x *axis.

We assigned common *cis*-elements in the miRNAtarget data to matrix models of transcription factor binding sites in JASPAR CORE database Ver.3 [29]. We also assigned *cis*-elements in promoters of human protein coding genes of DBTSS to JASPAR matrix models. Here, we used the jaspscan program (with 'matrix score' threshold set to ≥ 80) provided by EMBOSS-6.1.0 [30] for these assignments. Figure 3 shows two frequency distributions of the JASPAR matrix models to which we assigned the common *cis*-elements and *cis*-elements of DBTSS protein-coding gene promoters. The Kendall rank correlation test [31] revealed that the two distributions in Figure 3 were significantly correlated (*z*-score: +4.3), which indicates that there are no consensus sequences that are specific to common *cis*-elements.

### Predicting miRNA-target

We applied our method to 155 mature miRNAs in the prepared miRNA-target data. For comparative purposes, we also applied two existing methods, mi-Randa (Sep. 2008 Rel.) [32] and RNAhybrid Ver.2.1 [33], to the same data. These two methods search for potential binding sites in 3'UTRs of mRNAs using the seed region of a given mature miRNA according to specific base-pairing rules. Note that they do not rely on cross-species conservation of potential binding sites as in our method. However, they still have region-limited view of miRNA activity, that is, they do not take potential binding sites in coding exons into consideration. We applied the programs with default parameter sets. A threshold of RNAhybrid, 'minimum free energy', was ≤ −25.0. To test the programs, we applied the collection of 3'UTR sequences used in the miRNA-target prediction program, TargetScan Rel.4.0 [6]. To allow fair comparison with our method, we used only the 3'UTR sequences corresponding to all human protein coding genes of DBTSS (14,728 genes).

**Figure 4:**
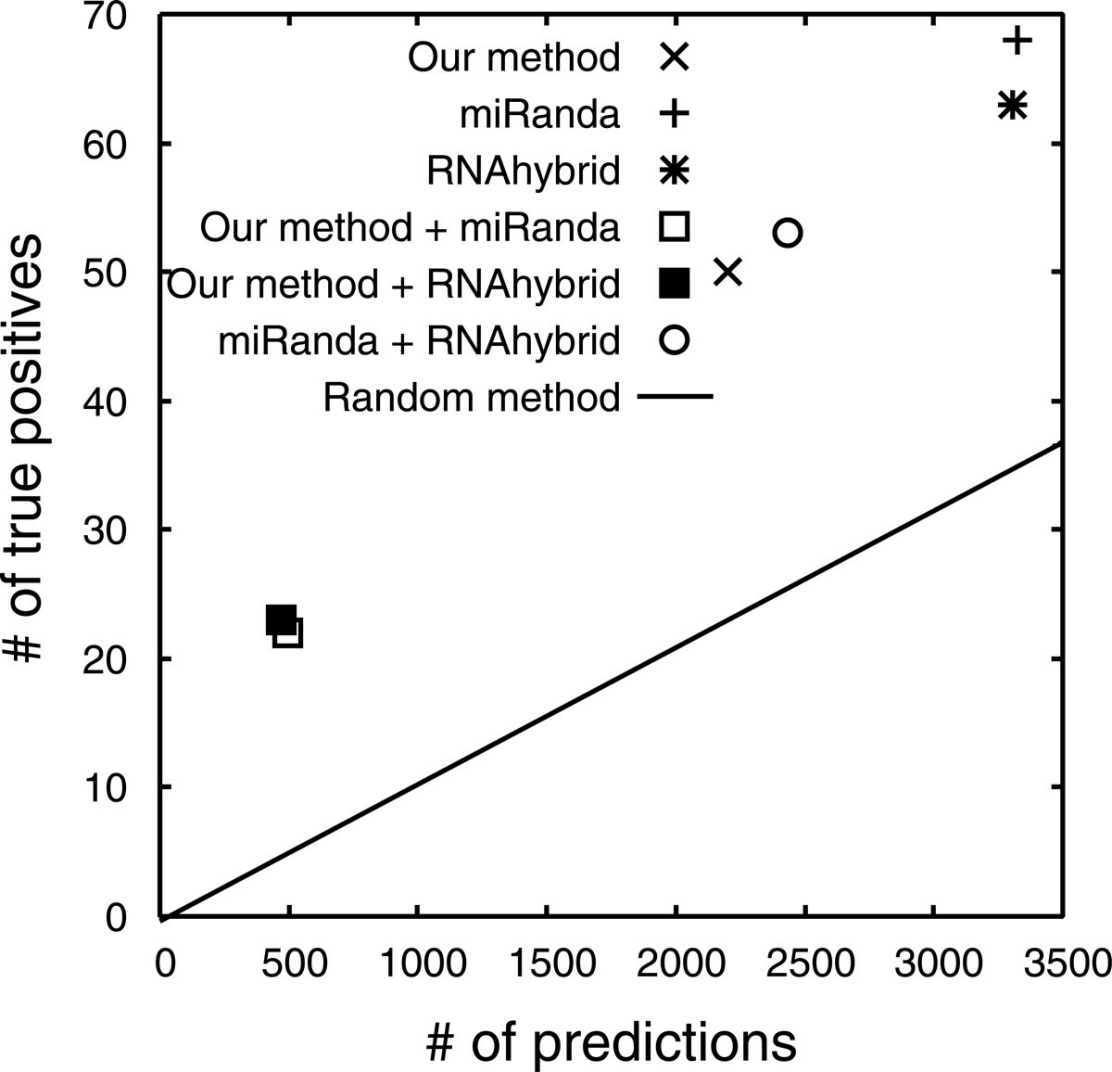
**Prediction accuracy of respective methods for the 155 miRNAs examined**. 'method A + method B' indicates a combination of method A and B. Combination results were calculated by intersecting the target genes predicted by A and B.

Figure 4 shows the prediction accuracy of the respective methods. Our method successfully predicted miRNA-targets in 50 of the data with an average of 2,204 predictions for each miRNA. These numbers will increase if we allow mismatches and gaps to find putative and common *cis*-elements in promoters (see 'Identifying putative *cis*-elements' and 'Detecting common *cis*-elements' in 'Methods' for detailed information). In contrast, miRanda predicted miRNA-targets in 68 of the data with an average of 3,332 predictions for each miRNA. RNAhybrid predicted miRNA-targets in 63 of the data with an average of 3,303 predictions for each miRNA. These numbers will increase if they focus on potential binding sites in coding exons of mRNAs as well as 3'UTRs.

#### It is possible to predict miRNA-targets based on common cis-elements

Although the predictive ability of our method is not particularly high, its prediction accuracy is comparable to that of miRanda or RNAhybrid (Figure 4). We evaluated statistical significances of their prediction abilities by using the binominal test [34], and found that *p *-value of our method was 5.69 × 10^−8 ^while those of miRanda and RNAhybrid were 1.09 × 10^−10 ^and 2.00 × 10^−8^, respectively. Those clearly show potential to predict miRNA-targets based on common *cis*-elements.

#### Advantages of our method

The main advantage of our method is that its prediction basis is significantly different from those of existing approaches. Combining existing methods results in only a minor decrease in both numbers of predictions and true positives, while a combination of existing methods and our method can drastically reduce both of these metrics (Figure 4). This is due to the different theoretical basis of the miRNA-target prediction; that is, our method focuses on promoter elements shared between miRNA and its target gene, while existing methods focus on miRNA-target binding sites. Our method provides a novel basis for miRNA-target prediction, which is entirely independent of cross-species conservation of miRNA-target binding sites. The data prepared contains 15 pairs of miRNA and its target gene the binding sites of which were known not to be conserved between related species [12]. Table 1 shows the prediction accuracy of our method for the 15 pairs. Our method correctly predicted 5 pairs, and its true positive rate (5/15) is comparable to that observed in Figure 4 (50/155). Moreover, while the existing methods focus on potential binding sites in 3'UTRs, not in coding exons, our method is entirely independent of their locations. Another advantage of our method is that it does not include learning steps. Thus, it does not require training data, and it is easy to apply it to other species. Although our method requires frequency distributions of the background incidence of common *cis*-elements, the same distributions should be applicable to other mammals whose genome compositions (e.g. GC contents) are similar to that of human.

**Table 1:** Prediction accuracy of our method for miRNA-target data whose binding sites are not conserved between related species.

miRNA	Target gene	Prediction
miR-375	C1QBP	×
miR-1	TIP120A	×
miR-1	PGM2	×
miR-1	SRXN1	×
miR-30a-3p	VEZATIN	×
miR-30a-3p	TMEM2	×
miR-30a-3p	CYR61	×
miR-30a-3p	TUBA3	×
miR-30a-3p	CDK6	×
miR-30a-3p	SLC7A6	×
miR-30a-3p	THBS1	×
miR-30a-3p	TMEM113	×
let-7b	KRAS	×
miR-23a	FLJ13158	×
miR-124	RELA	×

#### Disadvantages of our method

**Figure 5:**
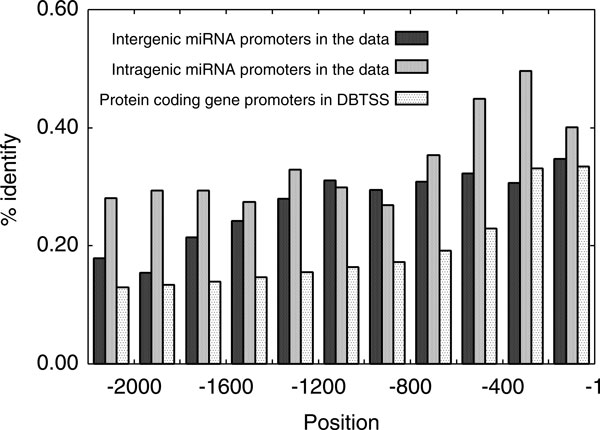
**Cross-species conservation of intergenic miRNA promoters**. Conservation was evaluated by using % identity in multiple sequence alignments between human, chimp, mouse, rat and dog. Multiple sequence alignments were obtained from the UCSC Genome Browser. For comparison, cross-species conservations of intragenic miRNA promoters and protein coding gene promoters are shown.

The main disadvantage of our method is that it includes promoter determination steps. This disadvantage is particularly an issue in cases of intergenic miRNAs that have their own promoters. Since currently available data of intergenic miRNAs are premiRNAs, not primiRNAs, TSSs cannot be exactly defined to determine their promoters. Thus, we simply designated their promoters as the genomic regions upstream from the 5' ends of the intergenic pre-miRNAs. This procedure occasionally fails to capture promoters, especially in cases of miRNAs containing introns. The reduced proportion of statistically significant common *cis*-elements in intergenic miRNA-target data (25/110) compared to intragenic miRNA-target data (32/97) may be a result of this issue. Thus, we examined the cross-species conservation of our intergenic miRNA promoter designations (Figure 5). Figure 5 shows the widespread moderate conservation over the [−1400, −1] region, where +1 is the 5' end of the intergenic pre-miRNAs. This indicates that our procedure captured the promoter in many cases. Nevertheless, it will be essential to accumulate intergenic pri-miRNA data to improve the accuracy of our method.

#### For which functional categories of target genes is our method effective/ineffective?

We checked Gene Ontology (GO) terms [35] of target genes by using Uniprot [36], and calculated the success rate of miRNA-target prediction by our method for every GO term. Since GO terms have hierarchical relationships with each other, we also checked all the parent terms, which are indirectly associated with GO terms of target genes. Table 2 summarizes lists of GO terms ranked according to the success rate of miRNA-target prediction by our method. That is, target genes which have these terms tend (not) to be co-regulated with corresponding miRNAs at the transcriptional level. A list of GO terms with high success rates (Table 2 upper) contained a high frequency of terms associated with regulation, response and development. These terms are consistent with the typical biological function of miRNAs. On the other hand, a list of GO terms with low success rates (Table 2 lower) contained a high frequency of terms associated with system process and cell cycle. System process represents a steady process. Cell cycle is a periodic biological process and also represents a steady process. Table 2 also provides information on the reliability of miRNA-target prediction by our method. If the predicted target genes have GO terms associated with regulation, response and development, then the prediction is considered reliable, whereas if the predicted target genes have GO terms associated with system process and cell cycle, then the prediction is considered unreliable.

**Table 2:** Lists of GO terms ranked according to success rate of miRNA-target prediction by our method.

Best 10		
Rank	GO term	Success rate (%)

1	Enzyme linked receptor protein signaling pathway	70.0 (7/10)
2	Negative regulation of cellular metabolic process	66.7 (8/12)
3	Sequence-specific DNA binding	62.5 (10/16)
4	Negative regulation of metabolic process	61.5 (8/13)
5	Response to stress	60.0 (6/10)
	Negative regulation of macromolecule metabolic process	60.0 (6/10)
7	Multicellular organismal development	59.4 (19/32)
8	System development	58.3 (14/24)
	Anatomical structure development	58.3 (14/24)
10	Regulation of developmental process	57.9 (11/19)

**Worst 10**		

Rank	GO term	Success rate (%)

1	System process	17.6 (3/17)
2	Neurological system process	20.0 (2/10)
	Cell cycle	20.0 (3/15)
4	Nucleoplasm	21.4 (3/14)
5	Nuclear lumen	22.2 (4/18)
6	Cell cycle process	23.1 (3/13)
	Cell cycle phase	23.1 (3/13)
8	Intrinsic to membrane	25.0 (7/28)
	Integral to membrane	25.0 (7/28)
10	Nuclear part	26.3 (5/19)

Success rate of miRNA-target prediction for a GO term *x *is given by 'number of true positives'/'number of target genes', where GO terms of the numerator and denominator are *x*.

#### Availability

All of the data described in this paper are available from the author on request. We applied our method to all human miRNAs in miRBase rel.12.0, and the results are also available.

## Methods

### Finding common cis-elements

We collected experimentally supported human miRNA-target data, and determined the associated promoter regions. Next, we identified potential *cis*-elements in each promoter based on cross-species conservation, and selected those that were common between the promoters of a particular miRNA and its target genes.

#### Collecting miRNA-target data

We collected a set of experimentally supported human miRNA-target data from TarBase ver.5.0 [22]. TarBase contains ~1,100 entries of human miRNA-target data, which comprise a collection of pairs of mature miRNAs and their target genes. From this data set, we selected 166 entries that had direct experimental support, e.g. reporter gene assay. By using miRBase rel.12.0 [5] and the UCSC Genome Browser [24], we identified genomic loci of the miRNAs and the target genes in the human genome (hg18). Since miRBase consists of pre-miRNA data, we assigned mature miRNAs in TarBase to pre-miRNAs of miRBase based on their names and sequences. In some cases, a mature miRNA was assigned to multiple pre-miRNAs. We discarded mature miRNAs that were not assigned to any premiRNAs. In summary, our filtered miRNA-target data set consisted of 71 mature miRNAs, 84 premiRNAs and 117 target genes. The data contained 155 pairs of mature miRNAs and their target genes, and 207 pairs of pre-miRNAs and their target genes.

#### Determining promoter regions

We classified miRNAs from the miRNA-target data into intragenic and intergenic subsets to identify their promoter regions. We searched for host genes whose genomic loci overlapped with those of the miRNAs on the same strands. Genomic loci of host genes were examined by using five human (hg18) gene annotation tracks (UCSC Genes, RefSeq Genes, human mRNA from GenBank, H-Invitational, and Ensembl Genes) from the UCSC Genome Browser. In cases where host genes were found, the corresponding miRNAs were classified as intragenic miR-NAs. The remaining miRNAs were classified as intergenic miRNAs. Six miRNAs (hsa-let-7a-3, hsalet-7b, has-mir-21, hsa-mir-24-2, hsa-mir-34a, hsamir-129-1) were classified as intergenic miRNAs despite their intersection with host genes, because the fractions of their overlap were relatively small. As a result, from the 207 pairs of pre-miRNAs and their target genes, 97 were classified as intragenic and 110 were classified as intergenic.

Intragenic miRNA promoters were defined as the genomic region −2000/+ 200 bp from the transcription start site (TSS) of the host gene (where +1 is TSS). Genomic locations of TSSs were obtained from DBTSS Ver.6.0 [23]. In cases where alternative TSSs were reported, we selected the TSS for which the 'Number of confident cDNAs' was maximal. If this number was small (≤ 3), we adopted the most upstream TSS provided either by RefSeq [37] or UCSC Genes.

Intergenic miRNA promoters were defined as the 2,200 bp genomic region upstream from the 5' end of the intergenic pre-miRNAs. In cases where the intergenic miRNAs form a cluster, we identified the most upstream miRNA within the cluster to assign a promoter of a polycistronic transcript. We regarded intergenic miRNAs as clustered, when distances to neighboring miRNAs were ≤ 5,000 bp

We defined the promoters of miRNA target genes using the same approach as that described for host genes above. We discarded coding regions from all promoters according to annotations of UCSC Genes.

#### Identifying putative cis-elements

We identified putative *cis*-elements in promoters of miRNA and target genes based on cross-species conservation. We first extracted the promoter regions from multiple sequence alignments of 28 vertebrate genomes as provided by the UCSC Genome Browser. Next, we identified ≥ 6 nt regions that were completely conserved between human, chimp, mouse, rat and dog, and defined these as putative *cis*-elements.

#### Detecting common cis-elements

By comparing putative *cis*-elements between promoters of a miRNA and its target gene, we searched for ≥ 6 nt identical subsequences, and defined these as common *cis*-elements. To evaluate the statistical significance of the subsequences, we determined the frequency distribution of common *cis*-elements that occur by chance alone by applying the following procedure. First, we prepared two sets of TSSs from DBTSS. The former consisted of TSSs whose promoters shows a cross-species conservation distribution similar to that of the miRNA promoters, while the latter consisted of TSSs whose promoters shows a cross-species conservation distribution similar to that of the target gene promoters. Next, we randomly selected a pair of TSSs: one from the former and the other from the latter. Then, we repeated this application 100,000 times. For each pair of TSSs, we determined promoter regions [−2000, +200], and detected common *cis*-elements according to the above procedure. Then, we recorded the frequency of their incidence for every sequence length. Finally, we summarized these for all pairs of TSSs, and obtained frequency distributions of common *cis*-elements that occurred by chance for every sequence length. The Bonferroni method was applied to correct for multiple testing [38]. A set of common *cis*-elements between promoters of a miRNA and its target gene was considered statistically significant where its occurrence distribution by chance was 5% or less.

### Predicting miRNA-target

We developed a method for miRNA-target prediction as described below. Note that the method does not rely on any features of binding sites in 3'UTRs and coding exons. (1) The method assigned a given mature miRNA to a pre-miRNA, and identified its genomic locus. Then, the method determined a promoter region of the pre-miRNA, and identified putative *cis*-elements. See 'Collecting miRNA-target data' ~ 'Identifying putative *cis*-elements' for detailed information. (2) For all protein coding genes of an organism from which the miRNA originates, the method determined their promoter regions, and identified putative *cis*-elements. Since we adopted human as a model organism, the method identified putative *cis*-elements in 14,728 promoters of all human protein coding genes from DBTSS. See 'Determining promoter regions' 'Identifying putative *cis*-elements' for detailed information. (3) For each of the protein coding genes, the method compared its putative *cis*-elements with those of the miRNA, and detected common *cis*-elements. Then, the method evaluated statistical significance of an occurrence distribution of the common *cis*-elements, and regarded a protein coding gene whose occurrence distribution was significant as a target. See 'Detecting common *cis*-elements' for detailed information. In step (1), a mature miRNA was sometimes assigned to multiple pre-miRNAs. In such cases, we applied the method to each of the pre-miRNAs, and took the union of all predicted target genes.

## Competing interests

The authors declare that they have no competing interests.

## Authors' contributions

The study was originally conceived and was designed by TY. TF prepared the data, implemented the method, and carried out the experiments. TF interpreted the results of the experiments, and wrote the initial draft of the manuscript. All authors revised and approved the final manuscript.

## Acknowledgements

This work was supported by KAKENHI (Grantin-Aid for Scientific Research) No.221S0002 and No.22240032 from the Ministry of Education, Culture, Sports, Science and Technology of Japan.

## Declarations

The publication costs for this article were funded by the above grants.

This article has been published as part of *BMC Genomics *Volume 14 Supplement 2, 2013: Selected articles from ISCB-Asia 2012. The full contents of the supplement are available online at http://www.biomedcentral.com/bmcgenomics/supplements/14/S2.

## References

[B1] Zamore PD B H Ribo-gnome: the big world of small RNAs Science 2005 309 1519 1524 10.1126/science.1111444 16141061

[B2] Shyu AB Wilkinson MF van Hoof A Messenger RNA regulation: to translate or to degrade EMBO J 2008 27 471 481 10.1038/sj.emboj.7601977 18256698 PMC2241649

[B3] Rigoutsos I New tricks for animal microRNAs: Targeting of amino acid coding regions at conserved and nonconserved sites Cancer Res 2009 69 3245 3248 10.1158/0008-5472.CAN-09-0352 19351814

[B4] Pasquinelli AE MicroRNAs and their targets: recognition, regulation and an emerging reciprocal relationship Nat Rev Genet 2012 13 271 282 22411466 10.1038/nrg3162

[B5] miRBase http://www.mirbase.org/

[B6] Lewis BP Burge CB Bartel DP Conserved seed pairing, often flanked by adenosines, indicates that thousands of human genes are microRNA targets Cell 2005 120 15 20 10.1016/j.cell.2004.12.035 15652477

[B7] Xie X Lu J Kulbokas EJ Golub TR Mootha V Lindblad-Toh K Lander ES Kellis M Systematic discovery of regulatory motifs in human promoters and 3'UTRs by comparison of several mammals Nature 2005 434 338 345 10.1038/nature03441 15735639 PMC2923337

[B8] Tian Z Greene AS Pietrusz JL Matus IR Liang M MicroRNA-target pairs in the rat kidney identified by microRNA microarray, proteomic, and bioinformatic analysis Genome Res 2008 18 404 411 10.1101/gr.6587008 18230805 PMC2259104

[B9] Chang TC Mendell JT microRNAs in vertebrate physiology and human disease Annu Rev Genomics Hum Genet 2007 8 215 239 10.1146/annurev.genom.8.080706.092351 17506656

[B10] Stefani G Slack FJ Small non-coding RNAs in animal development Nat Rev Mol Cell Biol 2008 9 219 230 10.1038/nrm2347 18270516

[B11] Zhang C MicroRNomics: a newly emerging approach for disease biology Physiol Genomics 2008 33 139 147 10.1152/physiolgenomics.00034.2008 18303086

[B12] Sethupathy P Megraw M Hatzigeorgiou AG A guide through present computational approaches for the identification of mammalian microRNA targets Nat Methods 2006 3 881 886 10.1038/nmeth954 17060911

[B13] Farh KK Grimson A Jan C Lewis BP Johnston WK Lim LP Burge CB Bartel DP The widespread impact of mammalian MicroRNAs on mRNA repression and evolution Science 2005 310 1817 1821 10.1126/science.1121158 16308420

[B14] Kim SK Nam JW Rhee JK Lee WJ Zhang BT mi-Target: microRNA target gene prediction using a support vector machine BMC Bioinform 2006 7 411 10.1186/1471-2105-7-411 PMC1594580 16978421

[B15] Yousef M Jung S Kossenkov AV Showe LC Showe MK Naïve Bayes for microRNA target predictions-machine learning for microRNA targets Bioinform 2007 23 2987 2992 10.1093/bioinformatics/btm484 17925304

[B16] Robins H Li Y Padgett RW Incorporating structure to predict microRNA targets Proc Natl Acad Sci USA 2005 102 4006 4009 10.1073/pnas.0500775102 15738385 PMC554828

[B17] Kertesz M Iovino N Unnerstall U Gaul U Segal E The role of site accessibility in microRNA target recognition Nat Genet 2007 39 1278 1284 10.1038/ng2135 17893677

[B18] Wang X El Naqa IM Prediction of both conserved and nonconserved microRNA targets in animals Bioinform 2008 24 325 332 10.1093/bioinformatics/btm595 18048393

[B19] Gennarino VA Sardiello M Avellino R Meola N Maselli V Anand S Cutillo L Ballabio A Banfi S MicroRNA target prediction by expression analysis of host genes Genome Res 2009 19 481 490 19088304 10.1101/gr.084129.108 PMC2661810

[B20] O'Donnell KA Wentzel EA Zeller KI Dang CV Mendell JT c-Myc-regulated microRNAs modulate E2F1 expression Nature 2005 435 839 843 10.1038/nature03677 15944709

[B21] Marco A Konikoff C Karr TL Kumar S Relationship between gene co-expression and sharing of transcription factor binding sites in *Drosophila melanogaster* Bioinform 2009 25 2473 2477 10.1093/bioinformatics/btp462 PMC2752616 19633094

[B22] TarBase http://www.microrna.gr/tarbase

[B23] DBTSS home http://dbtss.hgc.jp/

[B24] The UCSC Genome Browser database http://genome.ucsc.edu/

[B25] Sethupathy P Megraw M Barrasa MI Hatzigeorgiou AG Computational Identification of Regulatory Factors Involved in MicroRNA transcription Lecture Notes in Computer Science 2005 3746 457 468 10.1007/11573036_43

[B26] Rodriguez A Griffiths-Jones S Ashurst JL Bradley A Identification of mammalian microRNA host genes and transcription units Genome Res 2004 14 1902 1910 10.1101/gr.2722704 15364901 PMC524413

[B27] Lee Y Kim M Han J Yeom KH Lee S Baek SH Kim VN MicroRNA genes are transcribed by RNA polymerase II EMBO J 2004 23 4051 4060 10.1038/sj.emboj.7600385 15372072 PMC524334

[B28] Lee Y Jeon K Lee JT Kim S Kim VN MicroRNA maturation: stepwise processing and subcellular localization EMBO J 2002 21 4663 4670 10.1093/emboj/cdf476 12198168 PMC126204

[B29] The JASPAR database http://jaspar.genereg.net/

[B30] EMBOSS homepage http://emboss.sourceforge.net/

[B31] Kanji GK 100 Statistical Tests 2006 London: SAGE Publications

[B32] John B Enright AJ Aravin A Tuschl T Sander C Marks DS Human MicroRNA targets PLoS Biol 2004 2 e363 10.1371/journal.pbio.0020363 15502875 PMC521178

[B33] Rehmsmeier M Steffen P Hochsmann M Giegerich R Fast and effective prediction of microRNA/target duplexes RNA 2004 10 1507 1517 10.1261/rna.5248604 15383676 PMC1370637

[B34] The R project for statistical computing http://www.r-project.org/

[B35] The Gene Ontology http://www.geneontology.org/

[B36] UniProt http://www.uniprot.org/

[B37] NCBI Reference Sequence (RefSeq) http://www.ncbi.nlm.nih.gov/RefSeq/

[B38] Miller RGJ Simultaneous Statistical Inference 1981 New York: Springer Verlag

